# Pulmonary arteriovenous malformation (PAVM) embolization: prediction of angiographically-confirmed recanalization according to PAVM Diameter changes on CT

**DOI:** 10.1186/s42155-021-00207-9

**Published:** 2021-01-18

**Authors:** Jihoon Hong, Sang Yub Lee, Jung Guen Cha, Jae-Kwang Lim, Jongmin Park, Jaehee Lee, Seung-Ick Cha, Chang-Ho Kim, Hyewon Seo

**Affiliations:** 1 Department of Radiology, Kyungpook National University Hospital, 130 Dongdeok-ro, Jung-gu, 41944 Daegu, Republic of Korea; 2 Department of Radiology, School of Medicine, Kyungpook National University, Daegu, Republic of Korea; 3 Department of Internal Medicine, School of Medicine, Kyungpook National University, Daegu, Republic of Korea

**Keywords:** Pulmonary arteriovenous malformation, Transcatheter embolization, Computed tomography

## Abstract

**Background:**

To assess pulmonary arteriovenous malformation (PAVM) recanalization after embolization based on PAVM diameter changes on computed tomography (CT), with pulmonary angiography used as a gold standard.

**Methods:**

A retrospective review was done of patients from 2008 to 2019 with a PAVM treated with endovascular embolization. The treatment outcome was determined by conventional angiography. Follow-up pulmonary angiography was performed when recanalization was suspected on CT, or embolization of all lesions in multiple PAVM patients could not be completed in a single session. Patients who had no preprocedural or follow-up CT were excluded. Draining vein, feeding artery, and venous sac diameter were measured on CT, and diameter reduction rates were compared with the widely-used, binary 70 % criteria.

**Results:**

Forty-one patients with 114 PAVMs were treated during the study period. Eight patients with 50 PAVMs met the inclusion criteria. Mean vein, artery, and venous sac diameter reduction rates were as follows: 59.2 ± 9.3 %, 47.5 ± 10.6 %, and 62.6 ± 13.2 %, respectively, in the occluded group and 5.4 ± 19.5 %, 11.3 ± 17.7 %, and 26.8 ± 14.2 %, respectively, in the recanalized group. The area under the receiver operating characteristic curves for PAVM recanalization for the draining vein was 1.00, showing a better result than the artery (0.97) and sac (0.99). Patients showed > 42 % draining vein diameter reduction in the occluded group and < 32 % in the recanalized group. The widely-used 70 % criteria showed low specificity for predicting recanalization (draining vein, 7.3 %; venous sac, 41.7 %) but 100 % sensitivity for both the draining vein and venous sac.

**Conclusions:**

The widely-used 70 % binary criteria showed limited performance in predicting outcomes in this angiographically-confirmed case series. Further investigations are warranted to establish a strategy for detecting recanalization after PAVM embolization.

## Background

Transcatheter embolization is the treatment of choice for pulmonary arteriovenous malformations (PAVMs) and has a high level of technical success (Faughnan et al. 2011; White et al. 1996). However, the recanalization rates in treated PAVMs have been reported to be approximately 3–49 % (Faughnan et al. 2004; Lee et al. 1997; Mager et al. 2004; Pollak et al. 2006; Prasad et al. 2004; Shimohira et al. 2015; Remy-Jardin et al. 2006).

Pulmonary angiography is considered as a gold standard for evaluating PAVM recanalization (Faughnan et al. 2011); however, due to its invasiveness, chest computed tomography (CT) is preferred as the follow-up modality in clinical practice. Several methods have been used to assess treatment outcomes in follow-up CT (Lee et al. 1997; Mager et al. 2004; Pollak et al. 2006; Prasad et al. 2004; Remy-Jardin et al. 2006; Milic et al. 2005; Hart et al. 2010; Brillet et al. 2007; Letourneau-Guillon et al. 2010; Trerotola and Pyeritz 2010; Woodward et al. 2013; Kajiwara et al. 2014; Rabellino et al. 2014; Conrad et al. 2015). Among them, the method of evaluating PAVM enhancement in contrast-enhanced CT has limitations due to serious artifacts caused by metallic embolic materials and an increase in radiation dose. Therefore, measuring the vessel diameter change of a PAVM has been the main method (Lee et al. 1997; Remy-Jardin et al. 2006; Milic et al. 2005; Brillet et al. 2007; Letourneau-Guillon et al. 2010; Trerotola and Pyeritz 2010; Woodward et al. 2013; Kajiwara et al. 2014; Rabellino et al. 2014; Conrad et al. 2015). Since Lee et al. (Lee et al. 1997) first proposed 70 % regression of the PAVM sac and draining vein as resolution, numerous studies have measured treatment outcome based on the 70 % criteria (Milic et al. 2005; Letourneau-Guillon et al. 2010; Trerotola and Pyeritz 2010; Woodward et al. 2013; Kajiwara et al. 2014; Conrad et al. 2015). In addition, a 30 % criteria has been widely used as an alternative in determining treatment outcomes (Remy-Jardin et al. 2006; Brillet et al. 2007; Rabellino et al. 2014). However, a recent angiographically-confirmed case series demonstrated low sensitivity and specificity for both the 70 % and 30 % criteria (Belanger et al. 2016). Nevertheless, more evidence on this is needed, as the 70 % criteria is still being customarily used in many studies (Lee et al. 2019; Mahdjoub et al. 2018; Stein et al. 2017). Therefore, the authors sought to retrospectively investigate with an angiographically-confirmed case series the vessel diameter changes of treated PAVMs using serial CT follow-up. Subsequently, the cut-off value of vessel reduction rates for detecting recanalization was compared with the existing 70 % criteria.

## Methods

### Patient enrollment

**Fig. 1:**
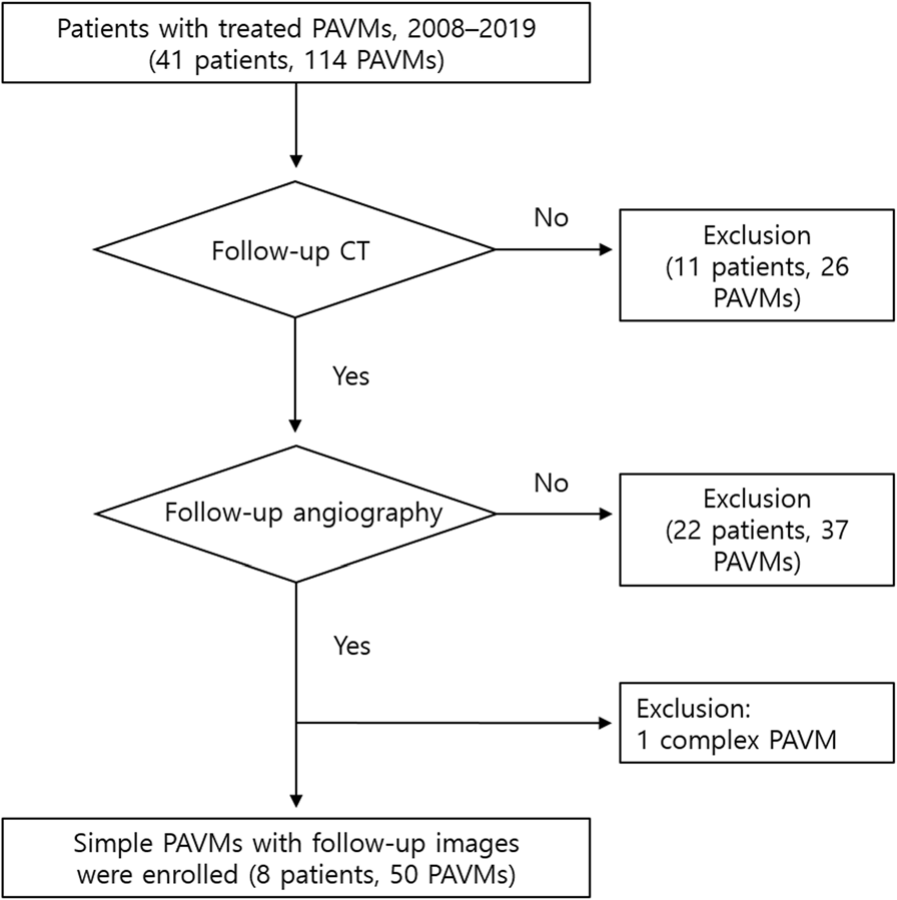
Flow chart showing patients enrolled according to inclusion and exclusion criteria

This retrospective study was approved by the hospital’s institutional review board, and informed consent was obtained from all patients at the time of the procedure. From August 2008 to November 2019, all patients from a database of subjects who underwent transcatheter embolization of PAVM in a referral hospital were included in the study. Inclusion criteria were as follows: (1) patients who receive transcatheter embolization of PAVM, (2) patients had an initial and follow-up CT before and after embolization, and (3) treated PAVMs had follow-up confirmative angiography. Patients were excluded if they had complex PAVMs with multiple feeding arteries. Patient enrollment is summarized in Fig. 1. Clinical histories, physical examinations, CT images, and interventional procedural images stored in the Picture Archiving and Communication System (PACS) and electronic medical records were reviewed.

### Embolization technique

After local anesthesia with 2 % lidocaine, venous access was obtained through the right common femoral vein. Subsequently, intravenous bolus administration of 3,000–5,000 U of heparin sodium followed. A 6-F guiding catheter (Envoy; Codman Neurovascular, Miami Lakes, Florida) was used in all cases. After right or left pulmonary angiography, the feeding artery was selectively catheterized with a 5-F hydrophilic catheter (Torcon NB Advantage; Cook Medical, Bloomington, Indiana) and coaxial microcatheter (Masters Parkway Soft; Asahi Intecc, Tokyo, Japan), if necessary. The type, size of vascular plugs (AMPLATZER Vascular Plugs [AVPs]; AGA Medical, Plymouth, Minnesota) and coils (Pushable: Tornado, Nester; Cook Medical, Bloomington, Indiana, Detachable: Concerto; Medtronic, Minneapolis, Minnesota), number of coils, and their deployment locations at the feeding artery or venous sac were determined by the operators from the selective angiography findings. In case of feeding artery embolization, the catheter was inserted into the feeding artery and advanced as close to the venous sac as possible. Feeding artery embolization was performed using an AVP oversized by 20–50 % or coils oversized by 20 % in relation to the feeding artery diameter. For venous sac embolization, a microcatheter was advanced into the venous sac via the feeding artery, and detachable coils oversized by 20–50 % in relation to the draining vein were deployed first. After a mesh frame of detachable coils was created to prevent migration, subsequent smaller pushable coils were deployed until there was complete cross-sectional occlusion of the venous sac and distal feeding artery. In all cases, completion digital subtraction angiography was performed after embolization to confirm vessel occlusion.

### CT Examination

Initially and as a follow-up exam, all patients underwent contrast-enhanced chest CT, including with multidetector row scanners. To prevent air embolism during the CT scan all PAVM patients were referred to a highly skilled intravenous access team. The region scanned extended from the cervicothoracic junction to the upper abdomen, and images were acquired during a single breath-hold. For the examination, a total 80–100 mL of contrast medium was administered intravenously at a rate of 1.5–2 mL/s. Contiguous thin-section transverse CT images of the area of interest were reconstructed at 2.5-mm slice thickness without intervals by using dedicated soft-tissue kernels.

Follow-up CT was performed at approximately 6 and 12 months after embolization and then every 2 years after that. Rarely, if treatment was not completed in a single session in patients with multiple PAVMs, follow-up was performed earlier to evaluate previously treated lesions prior to additional sessions.

### Angiographic confirmation

Follow-up angiography was performed in the following 2 situations. First, in case of the draining vein or venous sac size reduction of the PAVM was insignificant or enhancement in the venous sac persisted in the serial follow-up CT, the patient underwent angiography for suspicion of recanalization. Second, in patients with multiple PAVMs, embolization of all lesions could not be completed in a single session, and previously treated PAVMs were evaluated during subsequent embolization sessions. All pulmonary angiographies were first performed in the right or left pulmonary artery using an injector. Injection rates ranged from 10 to 15 mL/sec for volumes of 20–30 mL per injection, and frame rates were 3 frames per sec. For a more accurate evaluation, selective angiographies were followed at the segmental pulmonary artery with injection rates of 3–5 mL/sec for volumes of 10–15 mL or at its distal levels with careful manual injection.

Recanalization of the PAVM was evaluated during angiography and defined as a case where the feeding artery, venous sac, and draining vein were simultaneously enhanced due to either: (a) restoration of blood flow through a previously treated feeding artery or (b) collateral perfusion via a pulmonary artery to pulmonary artery collaterals.

### CT Vessel Diameter analysis

On initial CT images, lobar location, type (simple or complex), and multiplicity of the PAVM were determined. Vessel diameters (draining vein, feeding artery, venous sac) were measured on initial and serial follow-up CT images by 2 radiologists. Measurements were made at the same location on all CT images, and the window level and width were unified to -500 and 2000. The boundary for pulmonary parenchyma was measured after sufficient magnification to clearly identify it. The feeding artery diameter was measured as close to the embolized segment as possible based on follow-up CT. The draining vein diameter was measured just distal to the venous sac before the confluence of adjacent parenchyma, avoiding any metal artifacts caused by the coils in the sac (Gamondes et al. 2016; Kawai et al. 2014). For the venous sac, the cross-sectional diameter of the thickest part perpendicular to the long axis was measured. However, when venous sac embolization was performed, the venous sac diameter could not be measured due to metal artifacts. The reduction rates of all vessel diameters were calculated by comparing the initial and last follow-up CT images using average values of measurements from 2 radiologists.

### Data analysis

All PAVMs were divided into occluded and recanalized groups according to the angiographic results. Differences in the initial vessel diameter, vessel size reduction rate, period of angiography and CT follow-up, embolic materials (AVP or coils), and embolization locations (feeding artery or venous sac) in the 2 groups were examined by the t-test or Fisher’s exact test. Interobserver agreement was examined by analyzing the intraclass correlation of data sets of vessel diameter values measured by 2 radiologists. A *p* value < 0.05 was considered statistically significant. Statistical analysis was performed by using statistical software (Medcalc, version 19.4.1; Mariakerke, Belgium).

The area under the curves (AUC) and optimal cut-off values of vessel size reduction rates for recanalization was obtained by receiver operating characteristic (ROC) curve analysis, and the sensitivity and specificity of the diagnosis was compared to the existing 70 % sac/vein size reduction criteria.

## Results

### Patient demographics

In total, 41 patients with 114 PAVMs were treated with transcatheter embolization during 54 treatment sessions. Among the 114 PAVMs, 26 lesions in 11 patients were excluded for lacking follow-up CT and 37 lesions in 22 patients lacking follow-up angiography. One complex PAVM with 2 feeding arteries was excluded. Finally, 8 patients (all female; mean age, 48 y, range 33–57 y) with 50 PAVMs who had follow-up CT and angiographic images were enrolled. Exclusion criteria and patient enrollment data are shown in Fig. 1.

**Fig. 2:**
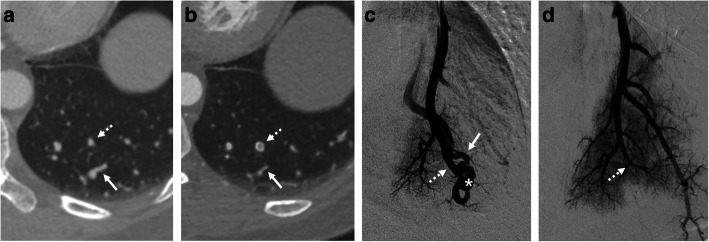
A 40-year-old female who underwent embolization 41 months earlier with a simple PAVM of the left lower lobe. Arrows, dashed arrows, and asterisks indicate draining vein, feeding artery, and venous sac, respectively. CT images obtained before (**a**) and after (**b**) embolization. The draining vein decreased from 2.4 mm to 1.1 mm after embolization (54.2 % reduction), and a vascular plug was deployed in the feeding artery. Occlusion was confirmed by pulmonary angiography obtained before (**c**) and after (**d**) embolization

**Fig. 3:**
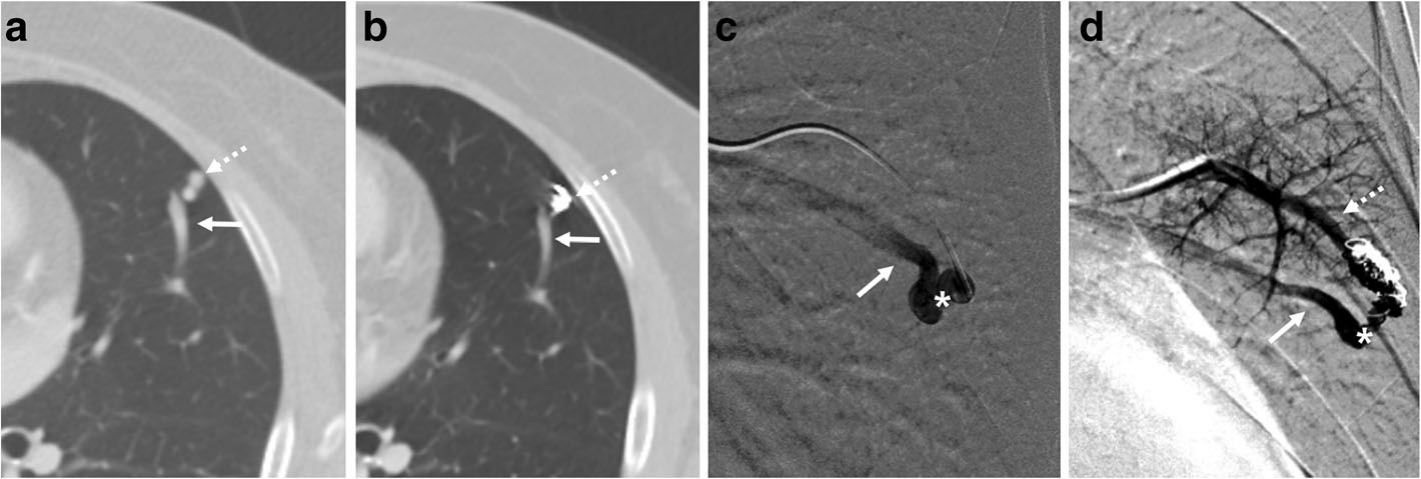
A 56-year-old female who underwent embolization 17 months earlier with simple PAVM of the left upper lobe. Arrows, dashed arrows, and asterisks indicate draining vein, feeding artery, and venous sac, respectively. CT images obtained before (**a**) and after (**b**) embolization. The draining vein decreased from 3.59 mm to 3.28 mm after embolization (8.6 % reduction) and coils were deployed in the feeding artery. Recanalization was confirmed by pulmonary angiography obtained before (**c**) and after (**d**) embolization

Of the 50 PAVMs, 44 (88 %) were angiographically evaluated in additional treatment sessions of multiple lesions, and in the remaining 6 (12 %), follow-up angiography was performed because recanalization was suspected in follow-up CT. Follow-up angiography revealed occlusion in 40 PAVMs (80 %) and recanalization in the remaining 10 (20 %) (1 was reperfused from an adjacent pulmonary artery) (Table 1). Figures 2 and 3 show representative cases with occluded and recanalized PAVMs confirmed by follow-up angiography.

**Table 1:** Patient demographic characteristics and numbers of treated PAVMs with follow-up CT and angiography

Pt. No.	Sex/Age (y)	Total No. of Treated PAVMs	Treatment Sessions	Treated PAVMs with Follow-up CT and Angiography	Angiographic Result	
					Occlusion	Recanalization
1	F/50	20	3	12	10	2
2	F/53	1	2	1	0	1
3	F/57	6	3	5	2	3^a^
4	F/49	12	3	10	8	2
5	F/56	1	2	1	0	1
6	F/40	3	2	1	1	0
7	F/33	8	2	4	4	0
8	F/44	20	4	16	15	1
Total		71	21	50	40	10

Note: *PAVM* pulmonary arteriovenous malformation

^a^One out of 3 lesions was reperfusion from pulmonary collateral

### PAVM and embolization characteristics

The level of embolization showed a significant difference in both groups (*p* = 0.010). In the angiographically-occluded group, feeding artery embolization was performed in 23 of 40 (57.5 %), and the remaining 17 PAVMs (42.5 %) were treated with venous sac embolization; whereas all 10 PAVMs (100 %) with recanalization were treated with feeding artery embolization. In total, 10/33 PAVMs treated with feeding artery embolization were recanalized, while 0/17 PAVMs treated with venous sac embolization were recanalized. Regarding embolic materials, a statistically significant difference was found in both groups (*p* = 0.11). Further, 36 % (9/25) of PAVMs treated with coils were recanalized, while 4 % (1/24) of PAVMs treated with AVP were recanalized. Among the patients who underwent venous sac embolization, coils were used in all 17 patients, and in one patient, an additional AVP was deployed. Meanwhile, out of 33 patients who underwent feeding artery embolization, 24 (72.7 %) had an AVP used and the remaining 9 (27.3 %) had coils used as the embolic material. No significant differences were observed between the 2 groups regarding the location of PAVMs and initial mean vessel diameters. The characteristics of treated PAVMs are summarized in Table 2.

**Table 2:** PAVM and embolization chracteristics of angiographically occluded and recanalized groups

Chracteristics	Occluded group (*n* = 40)	Recanalized group (*n* = 10)	*p* value
Lobar location (%)			0.116
Right upper lobe	1 (2.5)	0	
Right middle lobe	13 (32.5)	2 (20)	
Right lower lobe	10 (25)	1 (10)	
Left upper lobe	3 (7.5)	4 (40)	
Left lower lobe	13 (32.5)	3 (30)	
Mean draining vein diameter (mm)	3.63 ± 1.16 (1.63–7.97)	2.89 ± 0.71 (1.94–4.33)	0.062
Mean feeding artery diameter (mm)	3.25 ± 0.79 (2.24–5.42)	2.86 ± 0.50 (1.90–3.61)	0.142
Mean venous sac diameter (mm)	5.47 ± 1.76 (3.11–9.88)	5.08 ± 1.46 (3.33–7.35)	0.530
Embolization location (%)			0.010
Venous sac to feeding artery	17 (42.5)	0	
Feeding artery	23 (57.5)	10 (100)	
Embolic materials (%)			0.011
AVP	23 (57.5)^a^	1 (10)^b^	
Coils	16 (40)^a^	9 (90)	
Angiography follow-up (day)	501.2 ± 482.2 (35-1366)	896.6 ± 460.7 (75-1366)	0.024

Note: values presented as number (percentage) or mean ± standard deviation (range) where applicable

*AVP* Amplatzer vascular plug; *PAVM* pulmonary arteriovenous malformation

^a^One lesion was embolized with both AVP and coils

^b^Angiographically confirmed reperfusion

### Vessel diameter reduction

Vessel diameter changes in the occluded and recanalized group are summarized in Table 3. The vessel size reduction rate was calculated using the average of the data sets measured by 2 radiologists, and the agreement between the 2 readers was excellent. Intra-class correlation coefficients for draining vein, feeding artery, and venous sac were 96.4 %, 95.4 % and 97.1 %, respectively (*p* = 0.0001). Differences between the diameter reduction rates of the draining vein, feeding artery, and venous sac of the 2 groups were statistically significant (*p* < 0.0001).

**Table 3:** Comparison of draining vein, feeding artery and venous sac reduction rate between occluded and recanalized groups

	Occluded group (*n* = 40)	Recanalized group (*n* = 10)	*p* value
Draining vein reduction rate (%)	59.2 ± 9.3 (41.6–78.1)	5.4 ± 19.5 (-38.4-31.9)	< 0.0001
Feeding artery reduction rate (%)	47.5 ± 10.6 (14.6–65.7)	11.3 ± 17.7 (-20.5-36.0)	< 0.0001
Venous sac reduction rate (%)	62.6 ± 13.2 (41.3–79.5)	26.8 ± 14.2 (12.7–50.2)	< 0.0001
CT follow-up (day)	850.2 ± 508.0 (147–1613)	965.4 ± 385.5 (486–1411)	0.442

Note: values presented as mean ± standard deviation (range) where applicable

### Diagnostic criteria

**Fig. 4:**
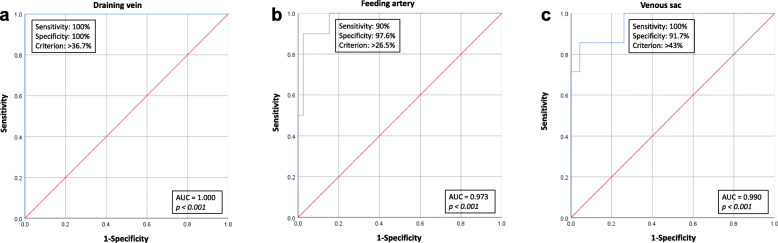
Receiver operating characteristic curve analysis for the draining vein (**a**), feeding artery (**b**), and venous sac (**c**) reduction rates

According to ROC curve analysis of the draining vein, feeding artery, and venous sac reduction rates, the AUC to detect PAVM recanalization were 1.000 (95 % CI 0.99-1.00), 0.973 (95 % CI 0.936-1.000), and 0.990 (95 % CI 0.859-1.000), respectively (Fig. 4). The cut-off values of each curve, which represents the optimal compromise between sensitivity and specificity, were 36.7 % (sensitivity 100 %, specificity 100 %), 26.5 % (sensitivity 90 %, specificity 97.6 %), and 43 % (sensitivity 100 %, specificity 91.7 %), respectively. According to the present data, the cut-off value of the existing < 70 % criteria to detect recanalized PAVMs showed specificity of 7.3 % in the draining vein and 41.7 % in the venous sac, with sensitivity of 100 % in both the draining vein and venous sac.

## Discussion

The present study analyzed an angiographically-confirmed PAVMs to validate the widely-used 70 % CT criteria. The interobserver agreement between the 2 radiologists for measuring vessel diameters were excellent. The optimal cut-off points of draining vein and venous sac reduction for detecting recanalization were different from the existing 70 % CT criteria conventionally used.

Various standards have been used in various institutions to evaluate the recanalization of treated PAVMs with follow-up CT (Lee et al. 1997; Mager et al. 2004; Pollak et al. 2006; Prasad et al. 2004; Remy-Jardin et al. 2006; Milic et al. 2005; Brillet et al. 2007; Letourneau-Guillon et al. 2010; Trerotola and Pyeritz 2010; Woodward et al. 2013; Kajiwara et al. 2014; Rabellino et al. 2014; Conrad et al. 2015). Among them, the 70 % venous sac or draining vein size reduction criterion has been most commonly used (Milic et al. 2005; Letourneau-Guillon et al. 2010; Trerotola and Pyeritz 2010; Woodward et al. 2013; Kajiwara et al. 2014; Conrad et al. 2015; Lee et al. 2019; Mahdjoub et al. 2018; Stein et al. 2017); however, there has been no standard for measuring vessel diameters or proposed time points for follow-ups; also, there has been no reliable background justification given for specifying the 70 % criteria. In one study, more than 70 % venous sac regression was noted in all angiographically-occluded PAVMs (Milic et al. 2005). However, in a recent study that analyzed 82 angiographically-confirmed PAVMs, the sensitivity of the 70 % size criterion was 98–100 % and the specificity was 20–47 % (Belanger et al. 2016). In addition, in a study by Kawai et al. (Kawai et al. 2014) comparing the shrinkage rate of the draining vein, time-resolved MR angiography (TR-MRA), and pulmonary angiography, 100 % sensitivity and 6 % specificity were shown when the 70 % standard was applied; and in their study, a 50 % criteria was suggested as the optimal cut-off. In the present study that analyzed 50 PAVMs, the sensitivity and specificity of the 70 % criteria were 100 % and 7.3–41.7 %, respectively. These results indicate the possibility of a high false-positive rate for the 70 % criteria and suggest that patients may be subjected to unnecessary additional examinations. If the existing 70 % criteria are lowered, the probability of missing PAVM recanalization associated with paradoxical emboli may increase. Nevertheless, according to the authors’ experience, the 70 % criteria is judged to be necessary to find a lower appropriate point because the gap with clinical practice is quite large. In the present study, the area under the ROC curve of draining vein reduction rate was 1.000, which showed a better result compared to other vessels, and the sensitivity and specificity for detecting recanalization of treated PAVMs at an optimal cut-off value of 36.7 % were 100 % and 100 %, respectively. This cut-off point was much lower than the existing 70 %.

Based on these results, the authors propose a new clinical strategy for evaluating treatment outcomes. Although the venous sac has been used the most as the standard of the existing size criteria, it is difficult to evaluate when performing venous sac embolization, which is increasingly used (Kajiwara et al. 2014). Therefore, the authors adopted the draining vein as a reference, which has a comparable AUC to other vessels in the present study and can be easily measured with high reproducibility (Gamondes et al. 2016). Though draining vein reduction rates above 42 % always indicated occlusion and below 32 % always indicated recanalization in this study, according to previous angiographically-confirmed studies (Belanger et al. 2016; Kawai et al. 2014), there was a relatively wide overlap between the 2 groups suggesting that the existing binary criteria is not suitable for clinical practice. Thus, the authors propose to introduce the concept of a “gray zone” for inconclusive results and recommend further work-up with TR-MRA as a non-invasive method rather than additional follow-up to prevent complications through early decision making. TR-MRA has proven its usefulness in evaluating the patency of PAVM in recent studies, and results that are comparable to conventional angiography have been reported (Shimohira et al. 2015; Kawai et al. 2014). The boundary of the gray zone is ideal to have an upper limit with little reduction in specificity while maintaining high sensitivity and a lower limit with little reduction in sensitivity while maintaining high specificity. When 60 % or more was defined as definite occlusion, a sensitivity of 100 % and 86–96 % and specificity of 44 % and 29–68 % were shown in the author’s and other angiographically-confirmed studies (Belanger et al. 2016; Kawai et al. 2014). On the other hand, when 20 % or less was defined as definite recanalization, a sensitivity of 75 % and 39 % and specificity of 100 % and 91 %, respectively, were shown in the studies (Belanger et al. 2016).

**Fig. 5:**
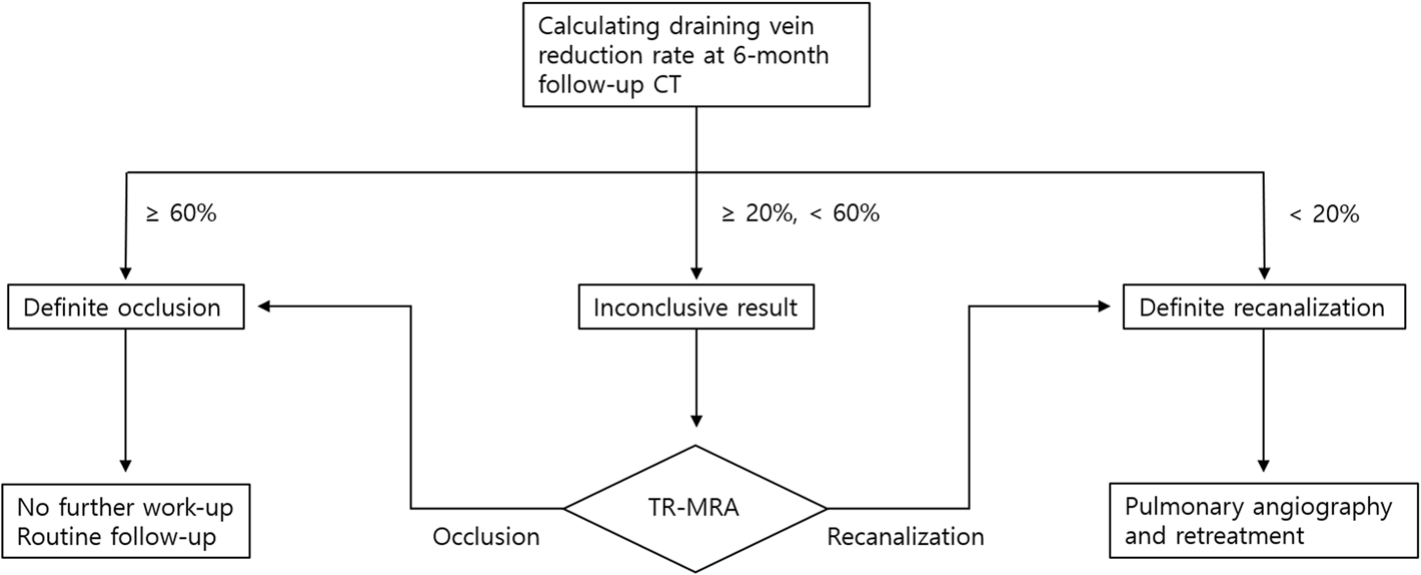
Flow chart of new clinical strategy for detecting recanalization. TR-MRA = time-resolved MR angiography

According to these results, based on the reduction rate of the draining vein on 6-month follow-up CT, treated PAVMs were divided into 3 categories as follows: (1) definite occlusion: if a reduction over 60 % is shown, judging that the treatment is appropriate, no further work-up is performed; (2) definite recanalization: in the case of under 20 % reduction, angiography and retreatment are performed assuming that shunt flow exists; and (3) inconclusive result: gray-zone between 20 % and 60 % is evaluated using TR-MRA to determine whether to recanalize. This strategy is expected to reduce unnecessary additional exams and contribute to the early detection of recanalization. A schematic diagram of the strategy is presented in Fig. 5.

The present study has some limitations. First, this study was a retrospective study and the sample size was relatively small due to it being conducted in a single institution with strict inclusion criteria. Second, the systemic collaterals were not evaluated. However, systemic reperfusion is less frequent and there are no complications due to a right-to-left shunt (Milic et al. 2005; Woodward et al. 2013). Additionally, since some of the treated PAVMs underwent follow-up angiography with suspicion of recanalization on CT and many other PAVM data were derived from patients with multiple PAVM, selection bias may have been introduced. Despite these limitations, in addition to the study of Belanger et al. (Belanger et al. 2016), this study provides more reliable evidence to suggest reassessing the existing 70 % criteria.

## Conclusions

The optimal cut-offs for predicting recanalization of the draining vein and venous sac were much lower than 70 %. The widely used binary 70 % criteria showed limited performance in predicting treatment outcome. Further investigations are warranted to establish a strategy suitable for clinical practice that detects recanalization after endovascular embolization of a PAVM.

## Data Availability

The datasets used and/or analysed during the current study are available from the corresponding author on reasonable request.

## Abbreviations

PAVM: Pulmonary arteriovenous malformation
CT: Computed tomography
AVP: AMPLATZER Vascular Plug
AUC: Area under the curve
ROC: Receiver operating chraracteristic
TR-MRA: Time-resolved MR angiography
